# Enhanced Photocatalytic Efficiency of N–F-Co-Embedded Titania under Visible Light Exposure for Removal of Indoor-Level Pollutants

**DOI:** 10.3390/ma8010031

**Published:** 2014-12-24

**Authors:** Seung-Ho Shin, Ho-Hwan Chun, Wan-Kuen Jo

**Affiliations:** 1 Department of Environmental Engineering, Kyungpook National University, Daegu 702-701, Korea; E-Mail: ssho@knu.ac.kr; 2 Department of Naval Architecture and Ocean Engineering, Pusan National University, 63 Jangjeon-dong, Geumjeong-gu, Busan 609-735, Korea; E-Mail: chunahh@pusan.ac.kr

**Keywords:** N,F co-embedment, N:F ratio, retention time, indoor air levels

## Abstract

N–F-co-embedded titania (N–F–TiO_2_) photocatalysts with varying N:F ratios were synthesized and tested for their ability to photocatalyze the degradation of pollutants present at indoor air levels using visible light. The synthesis was achieved using a solvothermal process with tetrabutyl titanate, urea and ammonium fluoride as sources of Ti, N and F, respectively. Three selected volatile organic compounds (toluene, ethyl benzene and o-xylene) were selected as the test pollutants. The prepared composites were characterized using X-ray diffraction, energy-dispersive X-ray spectroscopy, X-ray photoelectron spectroscopy and Ultra-violet (UV)-visible spectroscopy. The photocatalytic degradation efficiencies of N–F–TiO_2_ composites were higher than those obtained using pure TiO_2_ and N–TiO_2_. Moreover, these efficiencies increased as the N:F ratio decreased from sixteen to eight, then decreased as it dropped further to three, indicating the presence of an optimal N:F ratio. Meanwhile, as retention time decreased from 12.4 to 0.62 s, the average photocatalytic efficiencies decreased from 65.4% to 21.7%, 91.5% to 37.8% and 95.8% to 44.7% for toluene, ethyl benzene and o-xylene, respectively. In contrast, the photocatalytic reaction rates increased as retention time decreased. In consideration of all of these factors, under optimized operational conditions, the prepared N–F–TiO_2_ composites could be utilized for the degradation of target pollutants at indoor air levels using visible light.

## 1. Introduction 

Among semiconductors developed for photocatalytic applications, titanium dioxide (TiO_2_) is most common because of its oxidizing potential, chemical inertness and high photo-resistance [1,2]. Nevertheless, practical applications for TiO_2_ are limited by its wide band gap, which requires Ultra-violet (UV) radiation for photocatalytic activation [3]. As a result, much work has been directed at bringing the band gap energy difference to within the visible range throughout the use of metal [4,5] or non-metal [6,7,8,9] dopants. Certain metals, however, cause an increase in the number of recombination centers for photo-produced charge carriers, which results in thermal instability and a decrease in photocatalytic efficiency [10]. Furthermore, the toxic properties of heavy metals restrict later disposal.

Embedding TiO_2_ with non-metal elements, including nitrogen (N), fluorine (F), carbon (C) and sulfur (S), is therefore a promising alternative approach [6,7,8,9]. N doping especially has been extensively investigated, chiefly owing to the fact that the atomic size of N is comparable to that of oxygen (O), in addition to it forming metastable centers, exhibiting low ionization energy and having high thermal stability [2,10,11]. While the exact mechanism for the activation of N-doped TiO_2_ (N–TiO_2_) is uncertain, it is generally ascribed to the narrowed band gap resulting from the integration of N 2p states that are higher in energy than the top of the valence band [12,13]. Additionally, N doping alters the surface structure of TiO_2_ and controls the surface transfer of charge carriers, thereby enhancing photocatalytic performance [12,14]. Consequently, N–TiO_2_ possesses superior photocatalytic activity under visible light irradiation when compared to pure TiO_2_, allowing for the improved degradation of various pollutants, including aqueous rhodamine B, gaseous acetaldehyde and aromatic hydrocarbons [9,12,15,16].

In addition to N, F can increase the surface acidity of TiO_2_, thereby improving photocatalytic performance [17]. Certain studies reported that F doping alone does not significantly shift the light absorption into the visible spectral range [13], while other studies found that single modified titania with F could induce enhanced visible light-driven photocatalytic activity for the degradation of gas-phase acetone or acid orange 7 [18,19]. This difference is ascribed to different experimental conditions, such as the synthesis method, the F-to-TiO_2_ ratio, the target compound and the media. Regardless of this issue, in order to further take advantage of the benefits of N-doping, N–F-codoping of TiO_2_ (N–F–TiO_2_) has been developed [13,18,19,20]. When compared to single element doping, codoping strategies like this one that employed two elements, such as C and N [21] or Pt and N [22], have yielded higher photocatalytic activity.

Several studies have explored N–F–TiO_2_ synthesis, relying on sol–gel [18,19,20], solvothermal [13] and single-step combustion [23] methods, employing many different conditions by varying the calcination temperature and the ratio of the heteroatoms to Ti. However, the effect of the F to N ratio remains unaddressed. Additionally, previous studies have focused only on the photocatalytic efficiency of the degradation of specific aqueous pollutants; the reaction mechanisms for these degradations may be different outside of solution [24]. This study, therefore, addresses both of these issues, relying on a solvothermal route and analyzing the degradation of volatile organic compounds (VOCs) present at indoor levels under visible light irradiation. Pure TiO_2_ and N–TiO_2_ were also investigated for comparison. Toluene, ethyl benzene and o-xylene were chosen as the aromatic pollutants to be analyzed, because of their relatively high frequency in the selected environments [25] and the health hazards that they pose [26].

## 2. Results and Discussion

### 2.1. Characteristics of Prepared Photocatalysts

**Figure 1:**
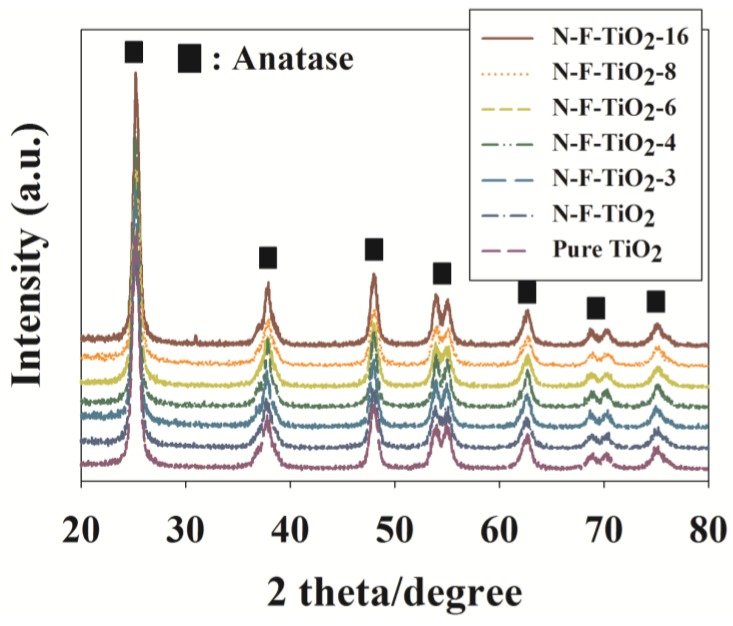
X-ray diffraction patterns of N–F–TiO_2_ with different N:F ratios (N–F–TiO_2_-3, N–F–TiO_2_-4, N–F–TiO_2_-6, N–F–TiO_2_-8 and N–F–TiO_2_-16), N–TiO_2_ and pure TiO_2_.

The N–F–TiO_2_ composites employing varying N:F ratios, along with the pure TiO_2_ and N–TiO_2_ reference catalysts, were characterized by X-ray diffraction (XRD), energy dispersive X-ray analysis (EDX), X-ray photoelectron spectroscopy (XPS) and UV-visible spectroscopy (UV-Vis). The corresponding XRD patterns are shown in Figure 1. The N–F–TiO_2_ composites, as well as the two reference photocatalysts, exhibited only anatase phase peaks at 2θ = 25.3°, 37.9°, 47.9°, 53.9°, 62.7° and 70.3°, consistent with previous studies [13,18], for samples calcined at or below 600 °C. Notably, both the N–F–TiO_2_ and N–TiO_2_ composites displayed a shift in the (101) crystal plane (2θ = 25.2°), suggesting the presence of lattice distortion [18]. Based on the anatase (101) diffraction data, the crystalline sizes of pure TiO_2_, N–TiO_2_ and N–F–TiO_2_ composites with N:F ratios of 16, 6, 6, 4 and 3 (referred to as N–F–TiO_2_-16, N–F–TiO_2_-8, N–F–TiO_2_-6, N–F–TiO_2_-4 and N–F–TiO_2_-3, respectively) were estimated to be 14.1, 12.6, 13.2, 13.4, 13.7, 13.8 and 14.0 nm, respectively. The smaller crystal sizes for the doped samples are in line with the proposal that doping might somewhat suppress TiO_2_ crystal growth [13].

**Figure 2:**
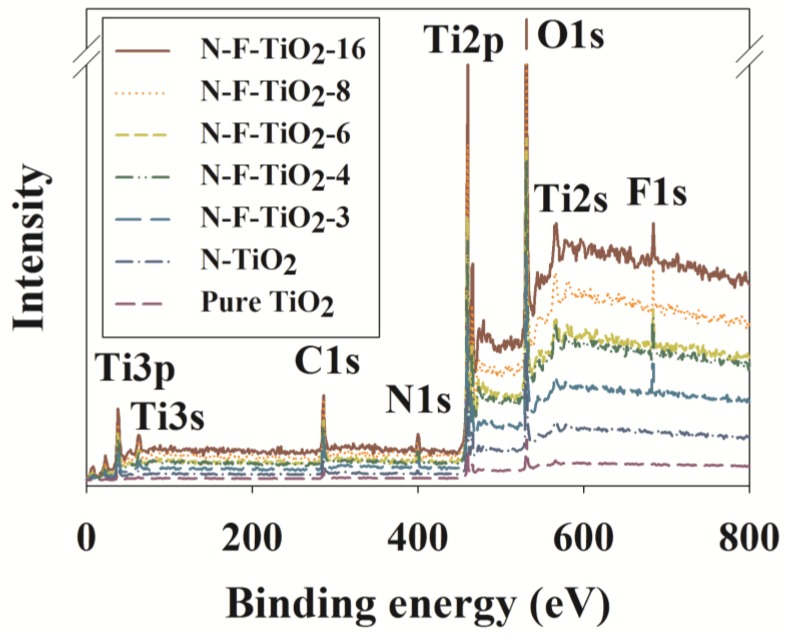
X-ray photoelectron spectroscopy of N–F–TiO_2_ with different N:F ratios (N–F–TiO_2_-3, N–F–TiO_2_-4, N–F–TiO_2_-6, N–F–TiO_2_-8 and N–F–TiO_2_-16), N–TiO_2_ and pure TiO_2_.

The chemical compositions of the photocatalyst samples were investigated with the help of XPS analyses. The XPS spectra confirmed the presence of both N and F in the N–F–TiO_2_ composites (Figure 2). Table 1 shows the derived binding energies and the N and F concentrations for all tested samples. F1s peaks appeared at 684.1, 683.1, 683.9, 683.1 and 683.9 eV in N–F–TiO_2_-16, N–F–TiO_2_-8, N–F–TiO_2_-6, N–F–TiO_2_-4 and N–F–TiO_2_-3, respectively; these peaks likely result from F^−^ ions adsorbed onto the TiO_2_ surface [20]. In addition, the F1s peak at 688 eV was assigned to F atoms that substituted for O sites within the TiO_2_ lattice (data not shown) [13]. Meanwhile, the N1s peaks were observed at 399.4, 399.2, 399.4, 399.4 and 399.4 eV for N–F–TiO_2_-16, N–F–TiO_2_-8, N–F–TiO_2_-6, N–F–TiO_2_-4 and N–F–TiO_2_-3, respectively; these were associated with molecularly chemisorbed N atoms [12]. The XPS data also revealed Ti2s, Ti2p, Ti3s, Ti3p and O1s peaks at 565.5‒567.2, 459.1‒459.9, 61.5‒62.9, 37.7, and 529.2‒529.8 eV, respectively. Pelaez *et al.* [20] have reported, based on XPS results, that N and F atoms can be successfully embedded into TiO_2_ using a fluorosurfactant-based sol-gel process. 

**Table 1:** Binding energy (eV) and amounts (%) of N and F for N–F–TiO_2_-3, N–F–TiO_2_-4, N–F–TiO_2_-6, N–F–TiO_2_-8, N–F–TiO_2_-16, N–TiO_2_ and pure TiO_2_ *.

Photocatalyst	Ti2p	Ti3p	Ti2s	Ti3s	O1s	N1s	F1s
N–F–TiO_2_-16	459.9	37.7	566.1	62.6	529.8	399.4 (7.2)	684.1 (0.4)
N–F–TiO_2_-8	459.9	37.7	567.2	62.6	529.4	399.2 (6.9)	683.1 (0.8)
N–F–TiO_2_-6	459.9	37.7	565.5	61.5	529.2	399.4 (7.0)	683.9 (1.3)
N–F–TiO_2_-4	459.7	37.7	566.2	62.6	529.2	399.4 (7.4)	683.1 (1.7)
N–F–TiO_2_-3	459.1	37.7	566.1	62.9	529.4	399.4 (6.8)	683.9 (2.1)
N–TiO_2_	459.1	37.7	567.1	62.6	529.8	NA	NA
pure TiO_2_	459.7	37.7	566.1	62.8	529.8	NA	NA

* Numbers in parenthesis represent the amounts (%) of N or F; NA, not available.

**Figure 3:**
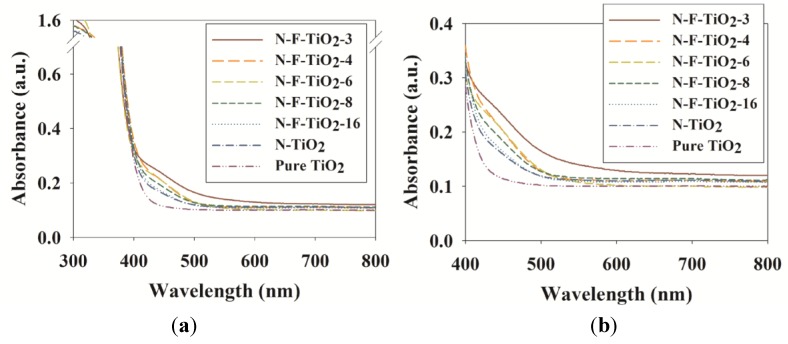
(**a**) UV-Vis spectra of N–F–TiO_2_ with different N:F ratios (N–F–TiO_2_-3, N–F–TiO_2_-4, N–F–TiO_2_-6, N–F–TiO_2_-8 and N–F–TiO_2_-16), N–TiO_2_ and pure TiO_2_; (**b**) The enlarged scale of the spectra is also provided.

Figure 3 displays the UV-Vis absorption spectra of the tested samples. Pure TiO_2_ showed an absorption edge at approximately 410 nm, a value that is in agreement with previous studies [15,27]. In contrast, the absorption spectra of both the N–TiO_2_ and N–F–TiO_2_ composites shifted toward the visible region, with values of 449.2, 456.4, 461.5, 471.1, 477.5 and 483.9 nm for N–TiO_2_, N–F–TiO_2_-3, N–F–TiO_2_-4, N–F–TiO_2_-6, N–F–TiO_2_-8 and N–F–TiO_2_-16, respectively. This effect was attributed to the impurity states at the substitutional lattice sites resulting from N integration [12,13,28]. Additionally, visible light absorption intensity was greater for N–F–TiO_2_ than for N–TiO_2_, while also increasing gradually as the N:F ratios decreased. Di Valentin *et al*. [19] also reported that the visible light absorption increased gradually as the N:F ratios decreased from to 100 to 1.0 in N–F–TiO_2_ composites, which were prepared by a sol–gel process. Both of these effects were attributed to some kind of synergistic effect, given that F alone usually does not derive efficient light absorption.

### 2.2. Photocatalytic Activities of N–F–TiO_2_, N–TiO_2_ and Pure TiO_2_

**Figure 4:**
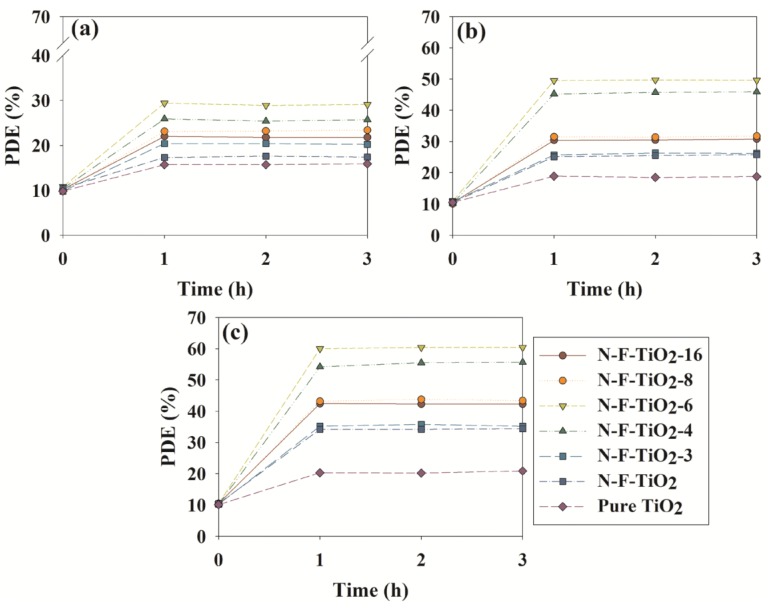
Time-series photocatalytic degradation efficiencies (PDEs, %) of (**a**) toluene, (**b**) ethyl benzene and (**c**) o-xylene as determined using N–F–TiO_2_ with different N:F ratios (N–F–TiO_2_-3, N–F–TiO_2_-4, N–F–TiO_2_-6, N–F–TiO_2_-8 and N–F–TiO_2_-16), N–TiO_2_ and pure TiO_2_.

The photocatalytic activities of the fabricated materials were investigated by exposure to visible light after allowing for adsorption in the dark. A control test performed using an uncoated Pyrex tube under visible light irradiation showed insignificant photolysis of the target compounds. Figure 4 shows time series of the photocatalytic degradation efficiencies (PDEs) of toluene, ethyl benzene and o-xylene for both reference photocatalysts and the assorted N–F–TiO_2_ composites under visible light exposure. N–F–TiO_2_ showed the highest activity, with average PDEs of 29.1%, 49.6% and 60.2% for toluene, ethyl benzene and o-xylene, respectively. N–TiO_2_ showed decreased activity of 17.4%, 25.3% and 34.2%, respectively, while pure TiO_2_ was the least active, with values of 15.7%, 18.7% and 20.4%, respectively. Previous studies have compared N–F–TiO_2_ performance with that of Degussa P25 TiO_2_, prepared TiO_2_, N–TiO_2_ and F–TiO_2_ and have demonstrated improved activity for the degradation of acetic orange, methyl orange methylene blue and microcystin in aqueous media [13,18,19,20,23]; again, this enhanced activity was ascribed to synergistic effects. 

Figure 4 also outlines the performance dependence on N:F ratios, with PDEs increasing as the N:F ratio decreased from sixteen to six. This pattern again suggests increasing synergy with increasing F content. However, the value then proceeded to drop as the N:F ratio decreased from six to three; this effect has been previously attributed to excess F species acting as an inhibitor by screening the TiO_2_ surface or capturing photon-generated holes [20]. Notably, N–F–TiO_2_-6 exhibited the highest PDEs, even though it absorbed less light than N–F–TiO_2_-4 and N–F–TiO_2_-3, suggesting that the photocatalytic activity is not strictly dependent on visible light absorption.

**Figure 5:**
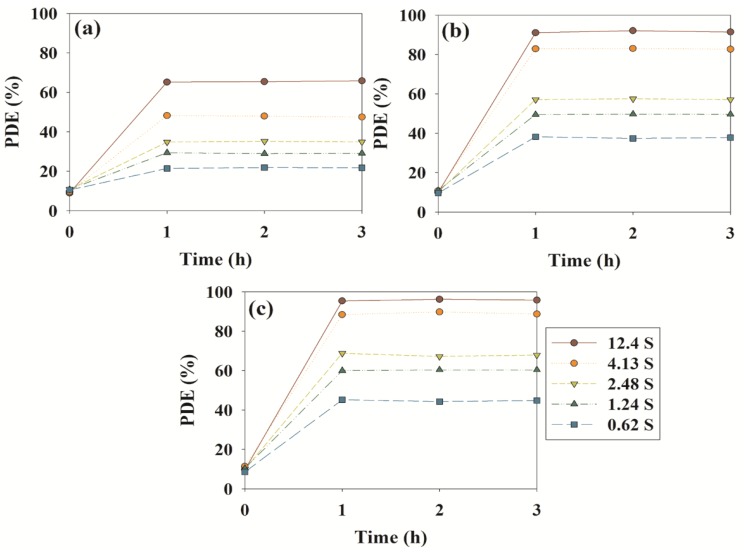
Time-series photocatalytic degradation efficiencies (PDEs, %) of (**a**) toluene, (**b**) ethyl benzene and (**c**) o-xylene as determined using N–F–TiO_2_-6, according to retention time.

Figure 5 shows time series of the PDEs for toluene, ethyl benzene and o-xylene obtained for N–F–TiO_2_-6 under visible light exposure based on retention time, demonstrating a positive correlation. Specifically, the average PDEs for toluene decreased from 65.4% to 21.7% as the retention time decreased from 12.40 to 0.62 s. This agrees with previous research by Jo and Kang [29], who reported that the PDEs of select aromatic vapors treated with polyacrylonitrile-supported TiO_2_ fibers decreased gradually with retention time. Retention times were estimated by dividing the reactor volume by the air flow rate. The low PDEs for low retention time conditions were ascribed to short reaction times inside the continuous-flow Pyrex reactor.

The photocatalytic reaction rates were estimated by combining the retention times with the following equation:

(1) $$r_{\text{R}}=f_{\text{c}}\cdot (C_{i}-C_{o})Q_{\text{air}}/A_{\text{c}}$$

where *r*_R_ represents the photocatalytic reaction rate (PRR) (μmol·m^−^^2^·s^−^^1^), *C_i_* and *C_o_* represent the upstream and downstream concentrations of each target chemical (ppm), respectively, *Q*_air_ represents the airstream flow rate (m^3^·s^−^^1^), *A*_c_ represents the inner-wall area coated with the photocatalyst (m^2^) and *f*_c_ represents the conversion coefficient (40.9 μmol·m^−^^3^·ppm^−1^). Unlike the PDEs, the PRRs increased as retention time decreased (Table 2), with values for toluene of 0.2 × 10^−3^ and 1.0 × 10^−3^ for retention times of 12.4 and 0.62 s, respectively. Previous studies reported the same pattern, suggesting that PRRs are affected by the mass transfer effect, a phenomenon that is closely associated with heterogeneous reaction kinetics [29,30]. Consequently, the dependence of PRRs on retention time was not assigned to photocatalyst surface reactions.

**Table 2:** Reaction rates (μmol·m^−2^·s^−1^) of three target compounds obtained using the N–F–TiO_2_-6 according to retention time.

Compound	Retention Time (s)				
	0.62	1.24	2.48	4.13	12.4
Toluene	1.0 × 10^−3^	0.7 × 10^−3^	0.4 × 10^−3^	0.3 × 10^−3^	0.2 × 10^−3^
Ethyl benzene	1.8 × 10^−3^	1.2 × 10^−3^	0.7 × 10^−3^	0.5 × 10^−3^	0.2 × 10^−3^
o-Xylene	2.1 × 10^−3^	1.4 × 10^−3^	0.8 × 10^−3^	0.5 × 10^−3^	0.2 × 10^−3^

## 3. Experimental Section

### 3.1. Synthesis and Characterization of Photocatalysts

N–F–TiO_2_ photocatalysts with varying N:F ratios were synthesized by a solvothermal method, using tetrabutyl titanate (TBT, Ti(OC_4_H_9_)_4_), urea (CO(NH_2_)_2_) and ammonium fluoride (NH_4_F) as sources of Ti, N and F, respectively. TBT (9 mL, 97%, Sigma-Aldrich, St. Louis, MO, USA) was added to ethyl alcohol (32 mL, 99.9%, Sigma-Aldrich) and concentrated nitric acid (0.4 mL, 69%, Merck, Whitehouse Station, NJ, USA). In addition, urea (0.22 g, 99%, Sigma-Aldrich), ammonium fluoride (98%, Sigma-Aldrich) and deionized water (2 mL) were added to ethyl alcohol (70 mL). The synthesis of N–F–TiO_2_-16, N–F–TiO_2_-8, N–F–TiO_2_-6, N–F–TiO_2_-4 and N–F–TiO_2_-3 required the use of 0.014, 0.028, 0.042, 0.056 and 0.084 g, respectively, of ammonium fluoride. Subsequently, the former solution was slowly added to the latter under magnetic stirring. After further stirring of the mixture at room temperature for 2 h, it was hydrothermally treated in an autoclave (150 mL) at 150 °C for 20 h. Finally, the treated mixture was washed with deionized water, dried at 100 °C overnight and treated at 400 °C for 3 h to obtain the desired N–F–TiO_2_ powder. Pure TiO_2_ and N–TiO_2_ were prepared following the same procedure, but without the addition of the corresponding element sources. It is worth noting that the so-called N:F ratio was only the ratio of precursors, which were highly unlikely to be the same as the composition of the resulting photocatalysts. The prepared photocatalysts were examined by XRD (Rigaku D/max-2500 diffractometer, Tokyo, Japan), XPS (PHI Quantera SXM, Chanhassen, MN, USA) and UV-Vis (Varian CARY 5G, Santa Clara, CA, USA).

### 3.2. Tests for Photocatalytic Activity 

The photocatalytic activities of the synthesized photocatalysts were tested using a plug-flow Pyrex reactor (3.8 cm i.d. and 26.0 cm length) with an inner wall coated in a thin film of the appropriate catalyst. To apply the coatings, titanium tetra-isopropoxide (50 mL, 97%, Sigma-Aldrich) was first added to glacial acetic acid (10 mL, 99%, Sigma-Aldrich) under stirring. The resulting solution was mixed with 1000 mL deionized water and then 10 mL nitric acid (98%, Sigma-Aldrich), stirred until a white precipitate was obtained and then heated at 80 °C for 5 h in a bath to obtain a sol. The selected, previously synthesized photocatalyst (2 g) was then added to the sol, after which the mixture was sonicated for 30 min to afford a sol coating. The outer wall of the Pyrex reactor was wrapped with a commercially-available vinyl sheet and dipped in the coating for 10 min, after which it was removed at a rate of 2 cm min^−1^ and kept in a clean room for 3 h. The coating and drying process was performed three times to maximize coating. A cylindrical lamp (F8T5DL, Youngwha Lamp Co., Seoul, Korea) designed to simulate daylight was placed in the coated reactor. A pure dried air stream provided from a compressed air tank was humidified by passing it through impingers, while the desired 0.1 ppm standard gas concentration was achieved by mixing the humidified air with the target chemicals, which were injected into a glass chamber via a syringe pump (Model Legato 100, KdScientific, Holliston, MA, USA). The prepared gas was routed into an empty buffering bulb (1 L) to minimize fluctuations in the supplied gas concentrations, after which it was fed into the reactor.

The photocatalytic decomposition efficiencies of the prepared photocatalysts were examined under a fixed stream flow rate of 1 L min^−1^ and a relative humidity of 45%, representative of a comfortable humidity level. The intensity of supplied light was 0.5 mW cm^−2^ at a distance from the lamp to the inner wall of the reactor. In addition, the PDE of N–F–TiO_2_-6, which was selected as representative of the N–F–TiO_2_ photocatalysts, because it showed the highest activity, was examined under retention times of 0.62, 1.24, 2.48, 4.13 and 12.4 s. All other parameters were adjusted to the values described above. Each experiment was conducted in triplicate.

Gas concentration measurements were completed upstream and downstream from the reactor. Samples were collected by drawing air from sampling ports fitted with Tenax adsorbent traps. Gases that had been adsorbed on the Tenax were pretreated using a thermal desorbing system (Perkin Elmer ATD 350, Llantrisant, UK) and analyzed by a gas chromatograph/mass spectrometer (Perkin Elmer Clarus SQ 8) outfit with a capillary column (DB-5, Agilent, Santa Clara, CA, USA). The target compounds were qualitatively determined on the basis of their retention times and mass spectra (Wiley 275 software library). Quantification of gaseous compounds was carried out using calibration curves, which were established using four concentrations normalized to an internal standard. Laboratory blanks and spiked adsorbent traps were used for the quality control of these analyses, with one blank trap analyzed on the day of the experimentation to check for any contamination. The detection limits of the target pollutants ranged from 0.002 to 0.005 ppm, depending on the chemical.

## 4. Conclusions

In this study, N–F–TiO_2_ photocatalysts with varying N:F ratios were synthesized and analyzed for their visible range photocatalytic performance in the degradation of VOCs present at standard indoor air concentrations. XPS demonstrated the successful integration of N and F into the TiO_2_, while UV-Vis spectra of both the N–F–TiO_2_ samples and the N–TiO_2_ control demonstrated improved visible light absorption. The N–F–TiO_2_ composites displayed superior photocatalytic degradation of toluene, ethyl benzene and o-xylene when compared to pure and N–TiO_2_, with precise activity dependent on the N:F ratio. In addition, retention time was found to be a significant factor affecting performance. Overall, these results indicate the utility of the prepared N–F–TiO_2_ composites under optimized operational conditions. 
